# Umbilical Cord PRP Accelerates Corneal Wound Healing via AQP1 Upregulation and Calcium Signaling

**DOI:** 10.3390/biology15080637

**Published:** 2026-04-17

**Authors:** Simona Martinotti, Giorgia Pellavio, Gregorio Bonsignore, Valeria Balbo, Laura Mazzucco, Umberto Laforenza, Elia Ranzato

**Affiliations:** 1 DiSIT—Dipartimento di Scienze e Innovazione Tecnologica, University of Piemonte Orientale, Viale Teresa Michel 11, 15121 Alessandria, Italy; simona.martinotti@uniupo.it (S.M.); elia.ranzato@uniupo.it (E.R.); 2 Laboratorio Integrato di Ricerca Preclinica, Azienda Universitaria Ospedaliera “SS Antonio e Biagio e Cesare Arrigo”, Via Venezia, 16, 15121 Alessandria, Italy; 3 Human Physiology Unit, Department of Molecular Medicine, University of Pavia, 27100 Pavia, Italy; giorgia.pellavio@unipv.it; 4 Transfusion Medicine and Regeneration Medicine, Azienda Universitaria Ospedaliera “SS Antonio e Biagio e Cesare Arrigo”, Via Venezia, 16, 15121 Alessandria, Italy

**Keywords:** aquaporin, calcium signaling, hydrogen peroxide, water permeability, proliferation, migration

## Abstract

Umbilical cord-derived Platelet-Rich Plasma (ucPRP) promotes corneal regeneration, yet the underlying cellular mechanisms remain incompletely understood. This study evaluated the effects of ucPRP on corneal epithelial cell physiology and wound healing dynamics. In scratch assays, ucPRP treatment significantly accelerated wound closure by enhancing cell migration and proliferation. Confocal microscopy using Fluo-3 demonstrated that ucPRP induces transient increases in intracellular calcium (Ca^2+^), suggesting the activation of upstream signaling events. Furthermore, treated cells exhibited increased membrane permeability to water and hydrogen peroxide (H_2_O_2_), which correlated with upregulation of Aquaporin-1 (AQP1). Since AQP1 facilitates cell motility, its upregulation links fluid transport mechanisms to the observed regenerative phenotype. These findings suggest that ucPRP drives corneal re-epithelialization by modulating Ca^2+^ signaling and increasing AQP1 expression, supporting its therapeutic utility in ocular surface repair.

## Simple Summary

The cornea is the transparent outer layer of the eye, essential for maintaining clear vision and protecting internal ocular structures. When this tissue is damaged by trauma or disease, incomplete healing can lead to permanent visual impairment. This study investigates the regenerative potential of a platelet concentrate derived from umbilical cord blood. Our laboratory tests on human corneal epithelial cells demonstrate that this biological product significantly accelerates the closure of surface wounds. This effect is achieved by activating intracellular calcium signaling and increasing the production of specific transport proteins called aquaporins, which facilitate the rapid movement of cells toward the injured area. These findings suggest that umbilical cord-derived plasma represents a promising natural therapy for treating severe ocular surface injuries, providing a more effective alternative to conventional eye drops for restoring corneal integrity and vision.

## 1. Introduction

The cornea, as the outermost transparent layer of the eye, plays a critical role in vision by providing a protective barrier against external insults and contributing significantly to light refraction [1].

Its remarkable transparency and precise curvature are essential for focusing light onto the retina, enabling clear vision. However, this delicate tissue is highly susceptible to various forms of injury, including mechanical trauma, chemical burns, infections, and degenerative diseases [2,3].

Damage to the corneal epithelium, if not properly addressed, can lead to chronic inflammation, persistent epithelial defects, stromal scarring, neovascularization, and ultimately severe visual impairment or even blindness [4,5]. Despite advancements in ophthalmology, current therapeutic strategies for corneal wound healing, such as topical medications, bandage contact lenses, or surgical interventions, often face limitations, including incomplete regeneration, the formation of opaque scars that compromise vision, and potential side effects [6]. Consequently, there is a pressing and unmet clinical need for novel, more effective, and truly regenerative approaches to corneal repair that can restore both structural integrity and functional transparency [7].

In recent years, regenerative medicine has explored the potential of biological products to enhance tissue repair. Platelet-rich plasma (PRP), an autologous blood product obtained by concentrating platelets from a patient’s own blood, has emerged as a promising therapeutic tool due to its rich content of growth factors, cytokines, and other bioactive molecules essential for tissue healing and regeneration [8,9,10]. These factors, released upon platelet activation, can stimulate cell proliferation, migration, angiogenesis, and extracellular matrix remodeling. While traditional PRP has shown efficacy in various clinical applications, the limitations of autologous collection (e.g., patient age, systemic conditions) have driven the search for alternative, allogeneic sources [11,12].

Umbilical cord-derived PRP (ucPRP) has garnered significant attention as a superior alternative, offering several distinct advantages [13,14]. ucPRP is readily available in abundant quantities from discarded birth tissues, possesses low immunogenicity due to its fetal origin, and contains a particularly rich repertoire of growth factors, making it an attractive candidate for regenerative therapies especially when conventional treatments fail [15]. Previous clinical reports have documented the successful treatment of patients with persistent corneal defects using ucPRP-based formulations [16].

Several clinical studies have reported that ucPRP eye drops or gels promote healing in persistent corneal ulcers, with high rates of epithelial closure and improvements in visual acuity. In prospective case series, complete healing rates ranged from 50% to over 80% depending on ulcer etiology, with most patients experiencing improvement and very few treatment failures. Notably, healing was often faster and more complete compared to conventional therapy alone, and adjunctive use with standard treatments further accelerated recovery [16,17,18].

The modulation of antioxidant systems and inflammatory cytokines plays a crucial role in protecting tissues from oxidative injury and promoting tissue repair [19]. Furthermore, natural bioactive compounds have been reported to enhance antioxidant defense mechanisms and reduce oxidative damage in various experimental models [20], suggesting that targeting oxidative stress is a key strategy in regenerative medicine. ucPRP contains high concentrations of growth factors (e.g., EGF, PDGF, VEGF) and anti-inflammatory cytokines, which stimulate corneal epithelial cell migration, proliferation, and tissue regeneration. Its unique immunomodulatory properties may also reduce inflammation and promote healing in cases where adult blood products are less effective or more inflammatory [16,17,18,21]. A single-center prospective study was conducted at the Transfusion and Regenerative Medicine Unit, SS. Antonio & Biagio and C. Arrigo Hospital (Alessandria, Italy), to evaluate the efficacy and safety of umbilical cord platelet-rich plasma in patients affected by ocular graft-versus-host disease following allogeneic hematopoietic stem cell transplantation and in those with Sjögren’s syndrome.

Treatment with heterologous ucPRP-based eye drops in 31 patients resulted in significant clinical improvement in 90% of cases, with a particularly notable enhancement in patients’ quality of life [22].

Despite these promising clinical outcomes, the cellular and molecular mechanisms underlying the regenerative effects of ucPRP on corneal epithelial cells remain poorly understood [16,23].

This study aims to comprehensively investigate the efficacy of ucPRP in promoting corneal cell repair through a series of in vitro experiments.

Our research specifically focuses on understanding the involvement of aquaporins (AQPs), a ubiquitous family of transmembrane water channel proteins that play crucial roles in maintaining cellular hydration, volume regulation, and rapid water transport across cell membranes. Besides their well-known roles, AQPs are also involved in other physiological processes, such as wound healing, where they help by promoting cell migration and proliferation [24,25,26]. Some AQP paralogs [27] facilitate hydrogen peroxide (H_2_O_2_) diffusion, which at physiological concentrations acts as a signaling molecule. Given the importance of AQPs in corneal transparency and cellular homeostasis, we explored their potential modulation by ucPRP during the complex processes of epithelial migration and wound closure. Furthermore, we investigated the dynamics of intracellular calcium variations, a key second messenger involved in regulating a multitude of cellular functions, including cell proliferation, migration, differentiation, and gene expression, all of which are vital for effective wound healing.

By examining these molecular and cellular events, we aimed to provide mechanistic insights into the regenerative effects of ucPRP and further clarify its potential as a therapeutic strategy for corneal wound repair.

## 2. Materials and Methods

### 2.1. Cell Culture

Human Corneal Epithelial Cells (hCEC) were utilized for all experimental procedures. These cells were acquired from healthy human corneas, obtained from Innoprot (Derio, Bizkaia, Spain). Upon arrival, hCEC were routinely maintained in a humidified incubator at 37 °C with 5% CO_2_ [28].

The cells were cultured in a specific corneal epithelial cell medium, supplemented with appropriate growth factors and antibiotics to ensure optimal proliferation and to prevent contamination (Corneal Epithelial Cell Medium Kit, Innoprot, Derio, Spain). Medium changes were performed every 2–3 days, and cells were passaged upon reaching 80–90% confluence using standard trypsinization protocols. For all experiments, cells between passages 3 and 7 were used to ensure consistent cellular characteristics and responsiveness.

### 2.2. Umbilical Cord-Derived Platelet-Rich Plasma Preparation and hCEC Cell Treatment

Following the donation and collection of umbilical cord blood in the delivery room, its cellularity was assessed to determine its intended use. If the cellularity (leukocytes) was ≤1.5 × 10^9^/mL, the blood could not, according to current regulations, be designated for banking to treat hematological disorders. Instead, it was allocated for the preparation of platelet concentrate (umbilical cord-derived Platelet-Rich Plasma for use in regenerative medicine or for research.

The procedure was as follows:

1. Centrifugation—Step 1: Centrifuge AFI-C300-R-Loreena (AFI Centrifuge, Château-Gontier-sur-Mayenne, France) the collection tubes for 10 min at 156× *g* (RCF) at 22 °C.
2. Transfer of Platelet-Rich Plasma (PRP) Under sterile conditions (BlueBeam 4 Class II Biological Safety Cabinet—Biohazard Cabinet, Blueair S.r.l., Capriolo (Brescia), Italy), transfer the Platelet-Rich Plasma (PRP) into anticoagulant-free tubes.
3. Centrifugation—Step 2: Centrifuge (Sintak AFI-C300-R-Loreena) the PRP for 10 min at 1203× *g* (RCF) at 22 °C.
4. Adjustment of Platelet Concentration: Under sterile conditions (BlueBeam 4 Class II Biological Safety Cabinet—Biohazard Cabinet), remove the excess Platelet-Poor Plasma (PPP) as needed to obtain PRP at an optimal platelet concentration of 1 × 10^9^/mL (±20%).
5. Platelet Resuspension: Gently resuspend the concentrated platelets in the remaining plasma volume until a homogeneous suspension is achieved.
6. Final Product: The Concentrated Platelet Unit is now ready for experimental use. The resulting product was then aliquoted and frozen at −40 °C and used for experimental purposes.

Treatments were performed by incubating hCEC cells with complete medium supplemented with ucPRP. An isotonic amount of sucrose was added to the control condition.

### 2.3. Scratch Wound Healing Assay

The migratory capacity of corneal cells was evaluated using a modified scratch wound healing assay [29]. Cells were seeded into 12-well tissue culture plates and allowed to reach approximately 90% confluence.

A sterile 200 μL pipette tip was then utilized to generate a single, linear scratch wound across the central region of each well. Any detached cells were subsequently removed by gently washing the wells twice with Phosphate-Buffered Saline (PBS). Following this, experimental treatments were introduced into their respective wells. Control wells received only complete culture medium.

Immediately after the scratch was created (defined as 0 h) and again at the 24 h time point, images of the scratch wound were captured using an inverted phase-contrast microscope equipped with a digital camera (Leica Microsystems, Wetzlar, Germany). The width of the scratch wound was measured using ImageJ software (NIH, Bethesda, MD, USA), a widely used image analysis tool [30]. Wound closure was then quantitatively assessed as the percentage of the initial wound area that had been covered by the migrating cells.

All experiments were conducted in triplicate and independently replicated at least three times to ensure reproducibility and statistical robustness.

### 2.4. Specific Inhibitors Utilized in the Study

A range of specific pharmacological inhibitors was utilized throughout this study to dissect the underlying signaling pathways involved. The following compounds were applied to functionally characterize the roles of key mediators: BAPTA-AM (30 μM, intracellular calcium chelator), SB203580 (20 μM, specific inhibitor of p38 mitogen-activated protein kinase (p38 MAPK)), PD98059 (10 μM, selective inhibitor of MEK1 and MEK2 (MAPK/ERK kinase 1 and 2)), U0126 (100 μM, selective inhibitor of MEK1 and MEK2 (MAPK/ERK kinase 1 and 2)), AG1478 (10 μM, selective inhibitor of the Epidermal Growth Factor Receptor (EGFR) tyrosine kinase), caffeine (10 µM, inhibitor of the IP3 receptor), U73122 (10 µM, phospholipase C (PLC) inhibitor), and suramin (100 µM, broad-spectrum antagonist of purinergic receptors).

### 2.5. Free Cytosolic Calcium Concentration ([Ca^2+^]_i_) Measurements

To quantify free cytosolic Ca^2+^, corneal cells were cultured on glass-bottom dishes (Iwaki Glass, Inc., Tokyo, Japan). Once sufficient cell attachment was achieved, cells were incubated in the dark for 30 min at 37 °C with 20 μM Fluo-3/AM [31]. This cell-permeant fluorescent calcium indicator was loaded using a previously established loading buffer.

Following the probe loading and subsequent washing steps, confocal time-lapse imaging was performed. Excitation was carried out with a 488 nm Argon laser, set at 0.5% power to minimize photobleaching, and emitted light was collected through a 505–550 nm bandpass filter [32].

A Zeiss LSM 510 confocal system, integrated with a Zeiss Axiovert 100 M microscope (Carl Zeiss Inc., Oberkochen, Germany), was utilized for data acquisition. Fluorescence signals from multiple cells were captured using a 20× objective lens, and the mean fluorescence intensity within regions of interest (ROI) was analyzed.

Fluo-3/AM calibration and the subsequent calculation of [Ca^2+^] were based on the Grynkiewicz equation [33]: [Ca^2+^] = Kd(Fmax − F)(F − Fmin).

Here, Kd was determined to be 400 nmol/L. The minimum (Fmin) and maximum (Fmax) fluorescence signals were established by sequentially treating cells with 500 μM A23187 for approximately 10 min, followed by 20 mM EDTA for 2 min.

### 2.6. Western Blot Analysis

The expression of AQP proteins and their changes were assessed by Western blot in hCEC with and without ucPRP treatment. Cells were homogenized with RIPA buffer as previously described in [27].

Protein concentration was measured by the Bradford method [34].

Cell homogenates were mixed with Laemmli buffer, and 30 μg of protein was resolved by SDS-PAGE and subsequently transferred to PVDF membranes, following the protocol described in [27]. Membranes were incubated for 1 h at room temperature in blocking buffer (5% skimmed milk, 0.1% Tween-20 in Tris-buffered saline) to prevent non-specific antibody binding.

The blots were incubated overnight at 4 °C with the blocking solution containing the antibodies listed in Table S1. The membranes were washed and incubated at room temperature for 1 h with a peroxidase-conjugated goat anti-rabbit antibody (AP132P; Merck, Milan, Italy), diluted 1:100,000 in blocking solution. Protein bands were visualized using the Westar Supernova chemiluminescence substrate (Cyanagen, Bologna, Italy). Molecular weights were determined by comparison with Precision Plus Protein Standards (Cat #1610394, BIO-RAD laboratories Srl, Segrate (MI), Italy). β2-microglobulin served as the housekeeping protein (Table S1). Signal detection was performed using the iBright CL1000 Imaging System (Thermo Fisher Scientific, Monza, Italy), and semiquantitative analysis was carried out with iBA software Version 5.5.0 (Thermo Fisher Scientific, Monza, Italy). The expression level of AQPs in ucPRP-treated and control hCEC cells was normalized and presented as the AQP/β2-microglobulin ratio.

### 2.7. Immunofluorescence

Immunolocalization of AQP1 was performed on sub-confluent hCEC cells untreated (control) and treated for 24 h with ucPRP grown on 18 mm × 18 mm glass coverslips.

Cells grown on coverslips were washed and fixed using 4% paraformaldehyde for 30 min, followed by washing with PBS. Antigen retrieval was performed by heating the submerged coverslips in retrieval buffer (10 mM citrate-HCl, pH 6.0) in a microwave for 10 min. Coverslips were then blocked with 3% bovine serum albumin (BSA) in PBS for 30 min at room temperature, and subsequently incubated overnight at 4 °C with the primary antibodies (listed in Table S1) diluted 1:100 in PBS. The slides were washed and then incubated at room temperature for 30 min with the secondary antibody Goat Anti-Rabbit IgG H&L (Alexa Fluor^®^ 488) pre-adsorbed (ab150081; Abcam, Cambridge, UK). Nuclei were counterstained with Hoechst (H3570, Thermo Fisher Scientific) and washed three times with PBS. The coverslips were mounted with the antifluorescence quenching FluoroSave reagent (345789, Merck Life Science S.r.l., Milan, Italy), and visualized using a Leica Thunder Imager DMi8 Microscope equipped with HC PL FLUOTAR 40×/0.8 objective (Leica biosystems, Buccinasco, Italy). The resulting images were quantified and analyzed utilizing the LAS AF Lite software (Leica Microsystems Application Suite Advanced Fluorescence Lite version 2.6.0, Buccinasco, Italy). To assess non-specific background signals, negative control experiments were included, involving cell incubation with nonimmune serum.

To compare immunofluorescence expression levels, consistent experimental conditions were maintained across samples, including the use of the same primary and secondary antibodies, staining protocols, and imaging parameters such as exposure time, gain, and laser intensity.

### 2.8. HyPer7 Plasmid Transfection, and Real-Time Imaging

Hydrogen peroxide diffusion rate was measured in hCEC treated with ucPRP for 24 h and compared to untreated controls. For this purpose, the hydrogen peroxide bioprobe HyPer7 targeted to the cytoplasm (pCS2 + HyPer7-NES) was used. The plasmid was generously provided by Vsevolod Belousov (IBCh, Moscow, Russia) (Addgene plasmid # 136467 and #136469). Specific details for plasmid #136467 are available via (http://n2t.net/addgene:136467, accessed on 14 April 2026) (RRID: Addgene_136467) [35].

hCEC cells were seeded in 2 mL Petri dishes to reach 60–70% confluence at the time of transfection. The HyPer7 bioprobe plasmid DNA (3 μg) was mixed with 200 µL of JetOPTIMUS buffer (# 717-60, Polyplus transfection, Illkirch-Graffenstaden, France) and 3 µL of JetOPTIMUS DNA Transfection Reagent (# 117-15, Polyplus transfection, Illkirch-Graffenstaden, France). After a 10 min preincubation at room temperature, the mixture was transferred into 2 mL of Opti-MEM™, and the transfection medium was subsequently added to the hCEC cells. Cells were incubated at 37 °C for 4 h in transfection medium, which was then replaced with Corneal Epithelial Cell Medium.

The H_2_O_2_ diffusion rate was measured 24 h after transfection by monitoring Hyper7 oxidation [27]. Following transfection, cells were maintained in a physiological medium for 10 min at ambient temperature as described in [27]. Then, 500 μL of hydrogen peroxide was added to the cells immersed in 2 mL of medium (final concentration, 50 μM). Images were acquired at 10 fps using a DMK 33UP1300 CCD camera and IC Capture software v2.5 (The Imaging Source, Visionlink, Seregno, MB, Italy). Image analysis was performed with ImageJ 1.54f software [34]. The time course of H_2_O_2_ diffusion was analyzed using a one-phase association equation in GraphPad Prism (v4.00, 2003) to determine the relative initial rate constant (k) and maximal fluorescence value (Y_max_).

### 2.9. Water Permeability Measurements

The osmotic water permeability of hCECs, either untreated or treated with ucPRP for 24 h, was studied using the stopped-flow light scattering technique. Measurements were conducted using an RX2000 stopped-flow apparatus (Applied Photophysics, Leatherhead, UK), which was equipped with a pneumatic trigger accessory (DA.1, Applied Photophysics, Leatherhead, UK) [36]. A Varian Cary 50 spectrophotometer (Agilent Technologies Italia S.p.A.,Cernusco sul Naviglio (MI), Italy) served as the detector. Changes in light scattering were monitored at a wavelength of 450 nm, achieving a dead time of 6 ms.

Cells were scraped from culture flasks, pelleted, and resuspended in PBS. The cell suspension was then mixed 1:1 (*v*/*v*) with ultrapure water, generating an osmotic gradient of 150 mOsm/L at room temperature. As a result, the cells swelled, and the corresponding decrease in light scattering was recorded for one minute at an acquisition rate of one reading every 0.0125 s. The time course of light-scattering changes was fitted to a one-phase exponential decay equation (GraphPad Prism v4.00, 2003) to determine the relative initial rate constant (k), an index of cell water permeability.

### 2.10. Statistical Analysis

Unless otherwise stated, data are presented as mean ± SD (standard deviation) of four independent experiments. Statistical analyses were conducted using GraphPad Prism 8 (GraphPad Software Inc., San Diego, CA, USA). The appropriate statistical tests were selected based on the experimental design and data distribution. Comprehensive statistical details, including sample size (*n*), the specific test employed, *p*-values, and the number of independent replicates, are explicitly provided within the respective figure legends.

## 3. Results

### 3.1. Umbilical Cord-Derived PRP (ucPRP) Promotes Dose-Dependent Wound Closure

**Figure 1:**
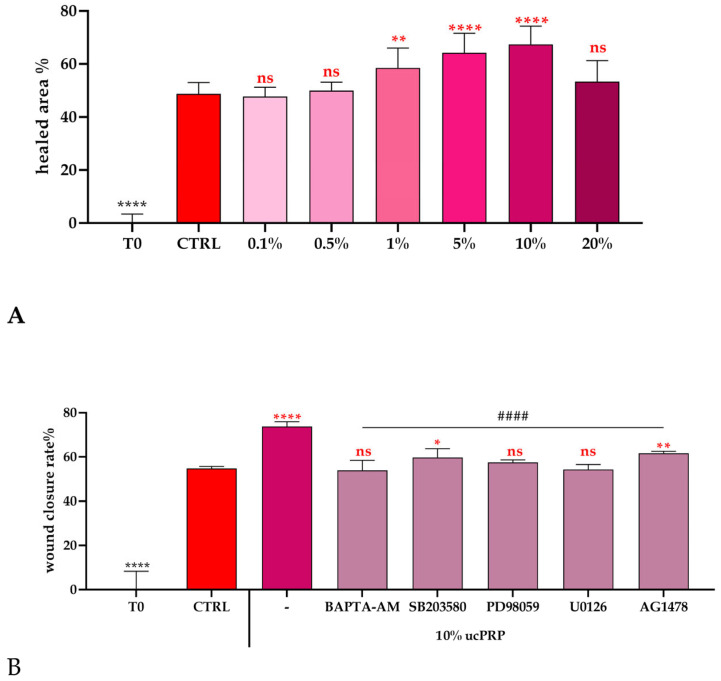
Dose-dependent and Mechanistic Effects of ucPRP on hCEC Wound Repair. (**A**) Dose–response relationship of ucPRP concentration and its impact on hCEC monolayer scratch wound closure. The rate of wound repair was calculated based on the reduction in wound width measured over a 24 h period. Cells designated as Control (CTRL) were cultured in normal growth medium. Results are expressed as the mean ± S.E.M. of wound closure, derived from 20 replicates across two separate experiments. Statistical differences between groups were determined by one-way ANOVA with Dunnet’s multiple comparison test (**** *p* < 0.0001; ** *p* < 0.01; ns: not significant). (**B**) This panel illustrates the role of specific signaling pathways in ucPRP-induced scratch wound repair. The wound closure rate was measured after 24 h of exposure to ucPRP in the presence of various inhibitors, including BAPTA-AM (30 μM), SB203580 (20 μM), PD98059 (10 μM), U0126 (100 μM), and AG1478 (10 μM). Data are presented as the mean ± S.E.M. and represent the percentage of wound closure inhibition, derived from two independent experiments, each encompassing 20 replicates. Statistical differences between groups were determined by one-way ANOVA with Tukey’s multiple comparison test (**** *p* < 0.0001, ** *p* < 0.001, and * *p* < 005 vs. ctrl; #### *p* < 0.0001 vs. ucPRP alone; ns: not significant).

Our investigations revealed that Umbilical Cord-Derived PRP effectively induces a dose-dependent wound closure, as visually represented in Figure 1A. This observation suggests a direct correlation between ucPRP concentration and its efficacy in promoting the healing process. However, our findings also reveal a plateau effect at the 20% concentration, indicating that this level is not optimal for maximal wound closure and that higher concentrations may not confer additional therapeutic benefits as already demonstrated for other cell lines [37,38,39,40,41,42].

Specifically, we found that increasing concentrations of ucPRP led to enhanced wound closure, indicative of accelerated tissue regeneration. Among the various concentrations tested, a 10% ucPRP concentration consistently demonstrated the most significant and optimal wound closure effects. Consequently, this concentration was selected for all subsequent studies to further elucidate the therapeutic potential of ucPRP.

Building upon our initial findings of optimal wound closure at 10% ucPRP concentration, the next crucial step was to evaluate the impact of various inhibitors to gain deeper mechanistic insights into how ucPRP promotes cellular mobility and, consequently, wound healing.

### 3.2. The MAPK/ERK Pathway Is a Key Mediator of ucPRP-Induced Wound Closure

We specifically investigated the effects of SB203580, PD98059, U0126, and AG1478. SB203580 is a specific inhibitor of p38 mitogen-activated protein kinase (p38 MAPK) [43], a signaling pathway involved in cellular stress responses, inflammation, and cell growth. PD98059 and U0126 are both potent and selective inhibitors of MEK1 and MEK2 (MAPK/ERK kinase 1 and 2) [44,45]. This pathway is a key component of the MAPK/ERK signaling cascade, which plays a central role in cell proliferation, survival, and differentiation. AG1478 is a selective inhibitor of the Epidermal Growth Factor Receptor (EGFR) tyrosine kinase [46], which is known to be involved in the activation of downstream pathways, including the MAPK/ERK pathway.

Our experiments (Figure 1B) revealed that in the presence of each inhibitor, the wound closure effect induced by 10% ucPRP was completely abolished. This indicates that the signaling pathways targeted by these inhibitors are all essential for the pro-healing effects of ucPRP. While all inhibitors were effective in blocking the wound closure, PD98059 and U0126 were the most effective in completely abrogating the ucPRP’s beneficial action. This result highlights the critical role of the MEK/ERK signaling pathway in mediating the cellular responses that lead to wound healing, suggesting it is a major downstream effector of the growth factors present in ucPRP. Although inhibitor-only controls were not included in the present experimental set, the concentrations used were previously validated in our earlier studies to not significantly affect cell viability or basal migration in the absence of stimulation [38,47]. Therefore, the observed inhibitory effects are likely attributable to the specific blockade of ucPRP-induced signaling rather than nonspecific or toxic effects of the compounds.

### 3.3. Intracellular Calcium Dynamics and ucPRP Response

Given the profound impact of BAPTA-AM—a calcium chelator—on the ucPRP-induced wound closure (Figure 1B), it became crucial to investigate intracellular calcium dynamics to further unravel the intricate mechanistic underpinnings of ucPRP’s effects. This observation aligns with previous studies showing that PRP’s pro-healing properties are highly dependent on calcium signaling in other cell lines [39,40,41,42,47,48]. For this investigation, we employed the highly sensitive fluorescent probe Fluo-3, which enabled real-time and precise monitoring of intracellular calcium fluctuations.

**Figure 2:**
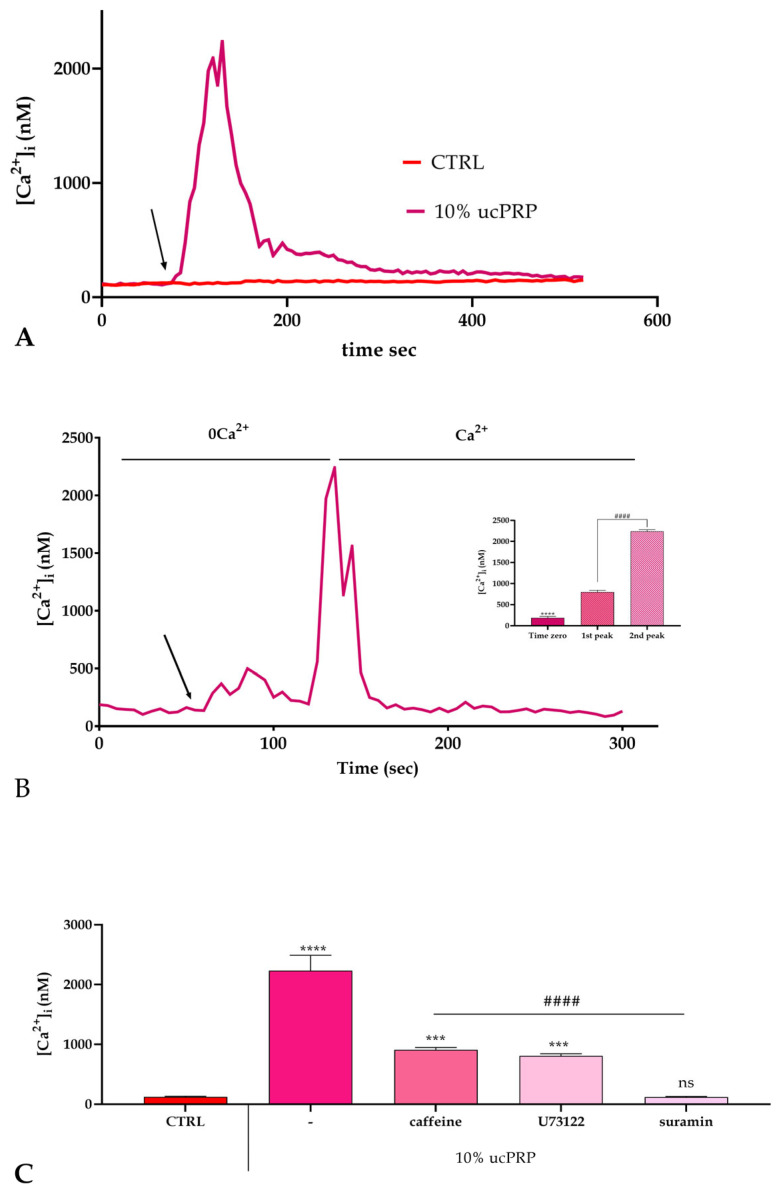
ucPRP induces a dose-dependent increase in intracellular Ca^2+^ concentration ([Ca^2+^]_i_) in hCEC cells. Panel (**A**) shows the [Ca^2+^]_i_ variations recorded at 5 s intervals. Control cells, incubated in confocal microscopy loading buffer, showed no significant [Ca^2+^]_i_ changes. In contrast, exposure to 10% ucPRP elicited a distinct pattern of Ca^2+^ signaling. The arrow indicates the addition of ucPRP. Data are presented as the mean ± S.E.M. of [Ca^2+^]_i_ traces from multiple cells. The number of cells analyzed were: 20 from 2 experiments for the control group, and 40 from 3 experiments for 10% ucPRP treatment. (**B**) To isolate the effect of intracellular Ca^2+^ mobilization, we stimulated hCEC cells with 10% ucPRP in the absence of external Ca^2+^ (0Ca^2+^). The subsequent reintroduction of external Ca^2+^ to the medium resulted in an increase in [Ca^2+^]_i_, highlighting a crucial role for extracellular calcium influx. Insert: Statistical differences between groups were determined using one-way ANOVA followed by Tukey’s multiple comparison test (#### *p* < 0.0001; **** *p* < 0.0001 vs. 1st and 2nd peak). (**C**) We measured the mean ± S.E.M. of the peak intracellular Ca^2+^ concentration ([Ca^2+^]_i_) response to 10% ucPRP under specific treatments. The data show the effects of various inhibitors on the Ca^2+^ response. We treated cells with ucPRP alone (20 cells from 3 experiments) or in combination with either 10 µM caffeine (40 cells from 3 experiments), 10 µM U73122 (*n* = 50 cells from 3 experiments), or 100 µM suramin. The asterisks on the bars indicate statistical significance as determined by one-way ANOVA with Tukey’s multiple comparison test (**** *p* < 0.0001 vs. ctrl; #### *p* < 0.0001 vs. ucPRP; *** *p* < 0.001 vs. suramin; ns: not significant).

Our experiment (Figure 2A) revealed a striking response upon the addition of ucPRP. We observed a marked and rapid response, characterized by a conspicuous and rapid peak in intracellular calcium concentration. This rapid surge in calcium suggests an immediate and robust cellular activation in response to the growth factors and signaling molecules present in ucPRP.

The robust, initial Ca^2+^ surge was notably transient, with intracellular calcium levels returning to baseline. This transient characteristic suggests the Ca^2+^ signal functions as a precise trigger, initiating specific cellular processes before homeostatic mechanisms restore resting conditions. This dynamic interplay of Ca^2+^ influx and subsequent efflux underscores its pivotal second messenger role, likely responsible for activating the complex cascade of proliferation and migration processes fundamental to effective wound healing.

To further characterize the origin of the [Ca^2+^]_i_ increase observed upon ucPRP exposure, we performed experiments under calcium-free conditions. The objective was to determine whether the calcium surge was due to influx from the extracellular environment or release from intracellular stores. As shown in Figure 2B, when the experiment was repeated in the absence of external calcium, we observed a significant reduction in the magnitude of the calcium peak following exposure to ucPRP. This finding suggests that a substantial portion of the intracellular calcium transient is dependent on calcium influx from the extracellular space. Furthermore, upon restoring calcium to the extracellular medium, the intracellular calcium peak was restored to its original high level. This dynamic confirms that extracellular calcium is a primary source for the ucPRP-induced calcium signal, playing a critical role in the cellular response.

To further elucidate the specific signaling pathways responsible for the ucPRP-induced calcium transient, we repeated the experiment in the presence of extracellular calcium but with various pharmacological inhibitors (Figure 2C). The inhibitors used were caffeine [49], U73122 [50], and suramin [51]. Caffeine is a known inhibitor of the IP3 receptor, which mediates calcium release from intracellular stores, particularly the endoplasmic reticulum. U73122 is a phospholipase C (PLC) inhibitor, which blocks the signaling cascade that leads to the production of inositol trisphosphate (IP3), a key second messenger for calcium release from intracellular stores. Considering the known importance of purinergic receptors on corneal cells [52], we specifically included suramin, a broad-spectrum antagonist of these receptors. It is well-established that purinergic receptors, particularly the P2Y2 subtype, are highly expressed on corneal epithelial cells and are essential for mediating cell-to-cell communication and migration required for wound healing [53]. Suramin is a broad-spectrum antagonist of purinergic receptors, which are activated by extracellular ATP and ADP and can lead to calcium influx and/or release.

The application of these inhibitors had a notable impact on the calcium peak. While caffeine and U73122 caused a reduction, suramin had the most significant effect, completely abrogating the ucPRP-induced calcium peak. The intracellular calcium levels in the presence of suramin were reduced to those of the untreated control. This result strongly suggests that the primary mechanism by which ucPRP elevates intracellular calcium is through the activation of purinergic receptors, and that calcium influx from the extracellular space, potentially through these channels, is the dominant event.

**Figure 3:**
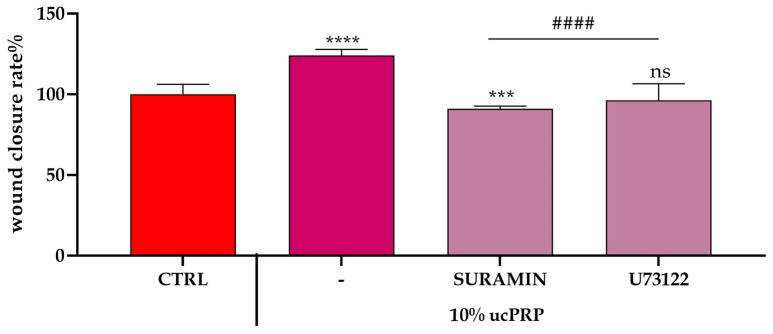
Inhibition of ucPRP-Induced Wound Repair in hCEC Monolayers. This panel illustrates the effects of specific chemical inhibitors on the ucPRP-induced scratch wound repair of hCEC monolayers. Wound closure efficacy was quantified as the difference in wound width observed between the 0 and 24 h time points. Control cells (CTRL) were maintained under normal growth conditions. Data are presented as the mean ± S.E.M. percentage of wound closure inhibition, derived from two independent experiments with 20 replicates each. Statistical differences were determined by one-way ANOVA with Dunnet’s multiple comparison test (**** *p* < 0.0001; *** *p* < 0.01 vs. ctrl; #### *p* < 0.0001 vs. ucPRP alone; ns: not significant).

To functionally validate the critical role of purinergic and PLC-dependent signaling in mediating the cellular effects of ucPRP, we repeated the wound closure experiments in the presence of suramin and U73122 (Figure 3).

Consistent with our calcium dynamics data, the application of both inhibitors had a significant negative impact on the ucPRP-induced wound closure effect. In particular, suramin, which almost completely abolished the intracellular calcium peak, also dramatically inhibited wound closure, reducing the rate to near-control levels. U73122, while less potent than suramin in blocking the calcium peak, also significantly impeded the wound closure process.

These results provide a crucial functional link, demonstrating that the signaling pathways targeted by suramin (purinergic receptors) and U73122 (PLC) are not only involved in the upstream calcium signaling but are also essential for the downstream cellular behaviors—namely, proliferation and migration—that drive effective wound healing. The data therefore confirm the critical importance of purinergic and PLC signaling as key mediators of ucPRP’s therapeutic effects on corneal epithelial cells.

### 3.4. ucPRP Treatment Increases Water and H_2_O_2_ Permeability in hCEC

Hydrogen peroxide (H_2_O_2_) is an important signaling molecule whose membrane permeability can be facilitated by aquaporins, and is involved in several cellular processes, including redox signaling and stress responses [27]. To assess whether ucPRP treatment affects H_2_O_2_ dynamics in hCEC, we evaluated intracellular H_2_O_2_ levels using the cytoplasmic ultrasensitive H_2_O_2_ indicator HyPer7 (pCS2 + HyPer7-NES) as a reporter.

**Figure 4:**
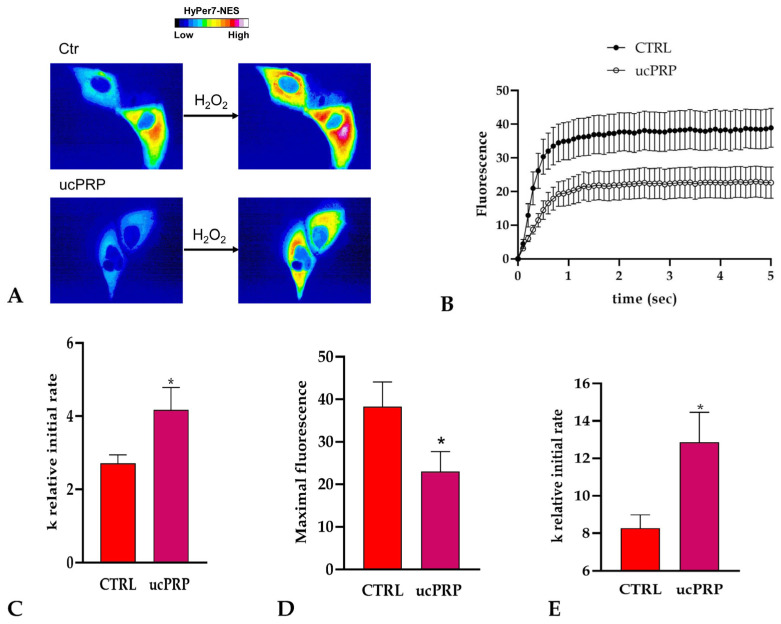
ucPRP treatment increased the hydrogen peroxide (**A**–**D**) and water permeability (**E**) of hCEC cells. (**A**) The left and right panels display representative frames extracted from time-lapse videos, illustrating the time course of H_2_O_2_ diffusion in untreated hCEC cells (CTRL) and hCEC cells treated with ucPRP (umbilical cord-derived PRP), respectively, both before and after the addition of 50 μM H_2_O_2_. The increase in HyPer7 fluorescence is shown in pseudocolor in the upper panel, with the scale indicated in the insert. (**B**) The time course of H_2_O_2_ fluorescence in both untreated (CTRL) and ucPRP-treated hCEC cells is presented, commencing immediately after the addition of 50 μM H_2_O_2_. Data represent the mean of at least three independent experiments. Panels (**C**,**D**) show the results of computerized least squares regression analysis (GraphPad Prism 4.00, 2003), employed to determine the relative initial rate values (k) (**C**) and the maximal fluorescence (Y_max_) (**D**). The experimental data points derived from the H_2_O_2_ time course curves were fitted using a one-phase exponential association equation. (**E**) Bars represent the osmotic water permeability of untreated (CTRL) and treated (ucPRP) hCEC cells expressed as k relative initial rate. Values are means ± SD of 4–15 single shots for each of 4–6 different experiments. * *p* < 0.05 compared to CTRL (Student’s *t*-test).

Treatment with ucPRP significantly decreased intracellular H_2_O_2_ levels in hCEC (Figure 4A,B,D), suggesting an increase in H_2_O_2_ permeability (Figure 4C).

Water permeability changes were evaluated using the stopped-flow light scattering method. As shown in Figure 4E, ucPRP treatment significantly increased water permeability by approximately 55%.

### 3.5. ucPRP Treatment Upregulates Aquaporin-1 Expression in hCEC Cells

**Figure 5:**
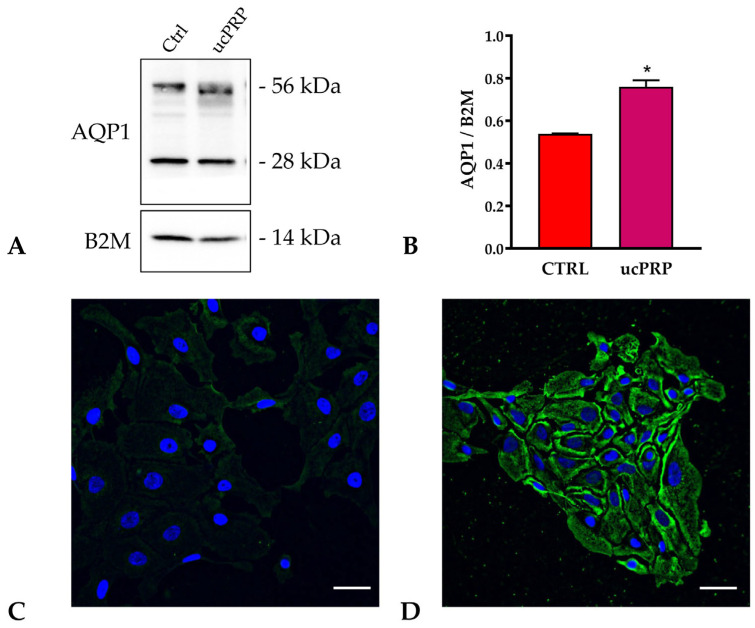
ucPRP treatment increased the expression of Aquaporin-1 protein. (**A**) Representative Western blots for aquaporin-1 (AQP1) and β-2-microglobulin (B2M) in hCEC cells untreated (Ctrl) and treated with umbilical cord-derived PRP (ucPRP). AQP1 protein levels were assessed, revealing two major bands at approximately 28 kDa (monomer) and 56 kDa (dimer). The intensity of these bands indicated a clear upregulation of AQP1 expression following ucPRP treatment. (**B**) Band densitometry was carried out with iBA software (Thermo Fisher Scientific, Milan, Italy) and normalized against B2M expression. * *p* < 0.05 compared to Ctrl (Student’s *t*-test). (**C**,**D**) Immunofluorescence images of AQP1 localization in hCEC untreated (**C**) and treated ucPRP (**D**). Green labeling indicates the presence of AQP1, more evident at the plasma membrane. Hoechst (blue) counterstained nuclei. Scale bar, 40 µm. The original western blot images are available in File S1.

Western blot experiments were conducted to understand which AQPs were affected by ucPRP treatment. AQP1, 3, 4, 5, and 9 were expressed at protein level in hCEC (Figure 5, Figures S1 and S2), while AQP6, 7, 8, and 10 were absent. The ucPRP treatment significantly increased the expression of AQP1 of about 40% (Figure 5A,B). The expression of the other AQPs was unchanged by the treatment (Figures S1 and S2). These results were further confirmed by immunofluorescence experiments in hCEC cells untreated and treated with ucPRP. Figure 5C,D, showed higher AQP1 expression in treated cells, mainly localized at the plasma membrane levels.

## 4. Discussion

This study provides compelling evidence for the multifaceted therapeutic potential of umbilical cord-derived Platelet-Rich Plasma in promoting corneal cell repair and regeneration. Our findings demonstrate that the application of ucPRP to corneal epithelial cells in a scratch wound assay significantly accelerated wound closure. This observed potentiation of wound healing is a critical step in restoring corneal integrity following various forms of injury or disease and is likely attributed to ucPRP’s potent stimulatory effects on both cell migration and proliferation, which are fundamental processes for effective corneal repair. However, although both processes are known to contribute to epithelial wound closure, the relative contribution of proliferation was not directly assessed in this study. In our experimental conditions, the rapid wound closure observed within 24 h suggests that cell migration may represent the predominant mechanism in the early phase of repair, although a contribution of proliferation cannot be excluded. The ability of ucPRP to encourage these cellular activities is crucial for quickly bridging gaps in damaged tissue and rebuilding the corneal surface.

The investigation into the underlying signaling mechanisms revealed that ucPRP’s effects are mediated through multiple pathways. The complete inhibition of wound closure by PD98059 and U0126 highlights the critical role of the MEK/ERK signaling pathway [54]. This is a well-established cascade that transduces extracellular signals, like those from the growth factors in ucPRP, into cellular responses related to proliferation and survival [55]. The effectiveness of AG1478 further suggests that ucPRP activates the Epidermal Growth Factor Receptor (EGFR), a known upstream activator of the MEK/ERK pathway, to initiate the healing cascade. The involvement of the p38 MAPK pathway, inhibited by SB203580, also points to a broader, coordinated cellular response to the complex mix of signaling molecules in ucPRP.

Intracellular calcium dynamics were identified as a pivotal second messenger event in response to ucPRP exposure. The observed transient and marked increase in intracellular Ca^2+^ was predominantly attributed to influx from the extracellular environment, evidenced by the significant attenuation of this signal upon extracellular Ca^2+^ depletion. This is further supported by the profound inhibitory effect of suramin, a purinergic receptor antagonist [56,57], which almost completely abolished the calcium transient. This suggests that purines, such as ATP and ADP, released from activated platelets in the ucPRP [58], bind to and activate purinergic receptors on corneal epithelial cells. This, in turn, triggers a rapid and robust calcium influx that is crucial for activating the downstream signaling cascades, including MEK/ERK. The lesser impact of U73122, a PLC inhibitor [59], suggests that while internal calcium stores may play a role, the direct influx mediated by purinergic channels is the dominant mechanism.

The functional validation of these findings through wound closure assays in the presence of suramin and U73122 provides a strong link between the molecular signaling events and the physiological outcome. The fact that both inhibitors significantly impaired wound closure underscores the necessity of both purinergic signaling and subsequent calcium transients for the successful regenerative effects of ucPRP.

Additionally, we observed that PLC inhibition with U73122 not only reduced the calcium peak but also significantly impaired wound closure. The epidermal growth factor (EGF), an important component of ucPRP, is a well-known activator of the Epidermal Growth Factor Receptor (EGFR). The activation of EGFR can lead to the production of diacylglycerol (DAG) through PLC. Our data suggest that ucPRP’s effects are not solely dependent on the IP3-mediated calcium release but also involve the parallel second messenger DAG, which is produced downstream of EGFR activation. This indicates a multi-faceted signaling network through which ucPRP promotes cellular healing.

Furthermore, our study uncovers a novel and significant mechanism by which ucPRP enhances corneal cell function: an increase in water and hydrogen peroxide permeability across corneal cells, which were directly linked to an upregulation of Aquaporin-1 (AQP1). AQP1, a vital water channel protein [60], plays a crucial role in maintaining osmotic balance and facilitating efficient water transport across cell membranes. The observed increase in AQP1 expression suggests that ucPRP actively influences the cellular machinery responsible for fluid dynamics, thereby optimizing the corneal microenvironment for effective healing and sustained functional integrity. This AQP1-mediated modulation of water permeability is critical for maintaining corneal integrity, as proper hydration and efficient solute exchange—key determinants of corneal transparency and physiological function—are prerequisites for robust and accelerated wound repair. Furthermore, AQP1 directly contributes to the healing process by promoting essential cellular activities, namely cell migration and proliferation [24,61]. Verkman and colleagues first demonstrated the involvement of AQP1 in wound healing [62]. The results presented here show that ucPRP treatment upregulated AQP1 supporting its involvement in wound healing. This is in accordance with previous findings obtained in murine and human cultured keratinocytes, which demonstrated the involvement of AQP1 in corneal wound healing [25,63].

AQP1 also functions as a peroxiporin, facilitating the diffusion of H_2_O_2_, as previously demonstrated in human lens epithelial cells [64]. Beyond its role in oxidative balance, H_2_O_2_ can act as a signaling molecule at physiological concentrations. In this context, it is known that the MEK/ERK pathway can be activated by redox-dependent mechanisms, including H_2_O_2_ signaling.

Based on these observations, it is plausible that ucPRP-induced upregulation of AQP1 may contribute to modulating intracellular H_2_O_2_ levels, which in turn could influence signaling pathways involved in wound healing. However, the present study does not directly demonstrate a causal link between AQP1-mediated H_2_O_2_ transport and ERK1/2 activation. Therefore, further studies will be required to clarify whether this mechanism contributes to the pro-healing effects of ucPRP. This pathway has been implicated in the regulation of cell proliferation and migration in different epithelial models, processes that are essential for wound closure [65,66,67,68].

## 5. Conclusions

**Figure 6:**
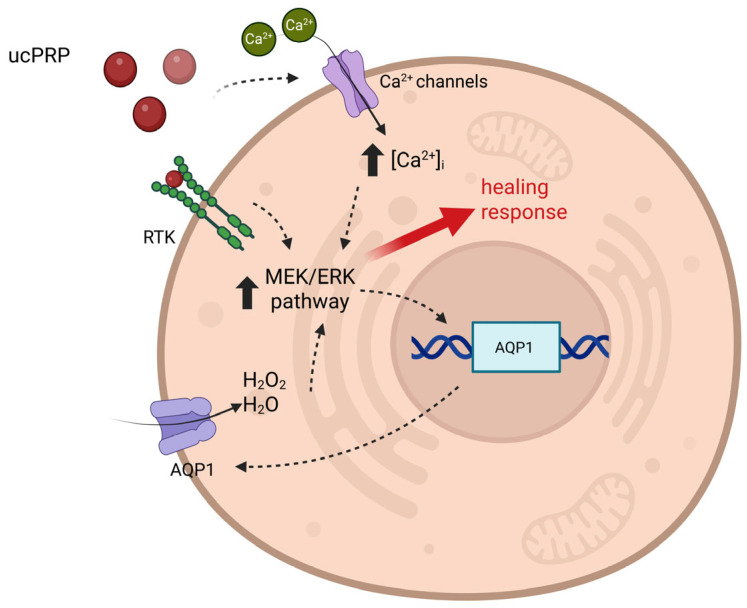
Diagram depicting the mechanism of action of ucPRP on hCEC cells, as characterized in the study. ucPRP Application: ucPRP, rich in growth factors and signaling molecules, is applied to the corneal cells. Purinergic Receptor Activation: Purines (e.g., ATP, ADP) released from the activated platelets in the ucPRP bind to and activate purinergic receptors on the cell surface. Increased Intracellular Calcium: This receptor activation causes a rapid and significant influx of calcium (Ca^2+^) from the extracellular environment. Activation of Signaling Pathways: The rise in intracellular calcium triggers a cascade of signaling events, including the activation of the MEK/ERK pathway. Upregulation of AQP1: The activation of these downstream signaling pathways stimulates the transcription and translation of the AQP1 gene, leading to an increase in the number of AQP1 channels on the cell membrane. Enhanced Permeability: The increased AQP1 expression not only facilitates water transport but also the diffusion of molecules like H_2_O_2_. The entry of H_2_O_2_ into the cell can further activate the MEK/ERK pathway, creating a reinforcing loop that strengthens the healing response. Created in BioRender (https://BioRender.com/21bt8g5, accessed on 2 February 2026).

In conclusion, the present study demonstrates that ucPRP promotes corneal epithelial wound healing in vitro through the activation of multiple intracellular pathways (Figure 6). These include the modulation of intracellular calcium dynamics and the upregulation of AQP1, which are associated with processes such as cell motility, proliferation, and membrane permeability to water and hydrogen peroxide.

However, the relative contribution of these processes under our experimental conditions remains to be fully elucidated, and no direct causal relationships between these pathways can be definitively established based on the current data.

Overall, ucPRP represents a promising candidate for corneal regeneration. Nevertheless, further studies, particularly in in vivo models, are required to confirm its therapeutic potential, clarify its mechanisms of action, and assess its safety and efficacy in clinical settings.

## Data Availability

Data presented in this study are available upon reasonable request from the corresponding author.

## Supplementary Materials

The following supporting information can be downloaded at: https://www.mdpi.com/article/10.3390/biology15080637/s1, Figure S1: Aquaporin-3, -4 expression was unchanged by ucPRP treatment; Figure S2: Aquaporin-5, -9 expression was unchanged by ucPRP treatment; Table S1: List of antibodies used in the study; File S1: Original Western blots.
