# Supplementary material for: Umbilical Cord PRP Accelerates Corneal Wound Healing via AQP1 Upregulation and Calcium Signaling
Source: Biology (Basel). 2026 Apr 17;15(8):637. doi: 10.3390/biology15080637 (PMC13113299; doi:10.3390/biology15080637)
Supplement: Supplementary file 1 [file biology-15-00637-s001.zip › File S1. Orignal WB images/Figure 5 original.pdf]

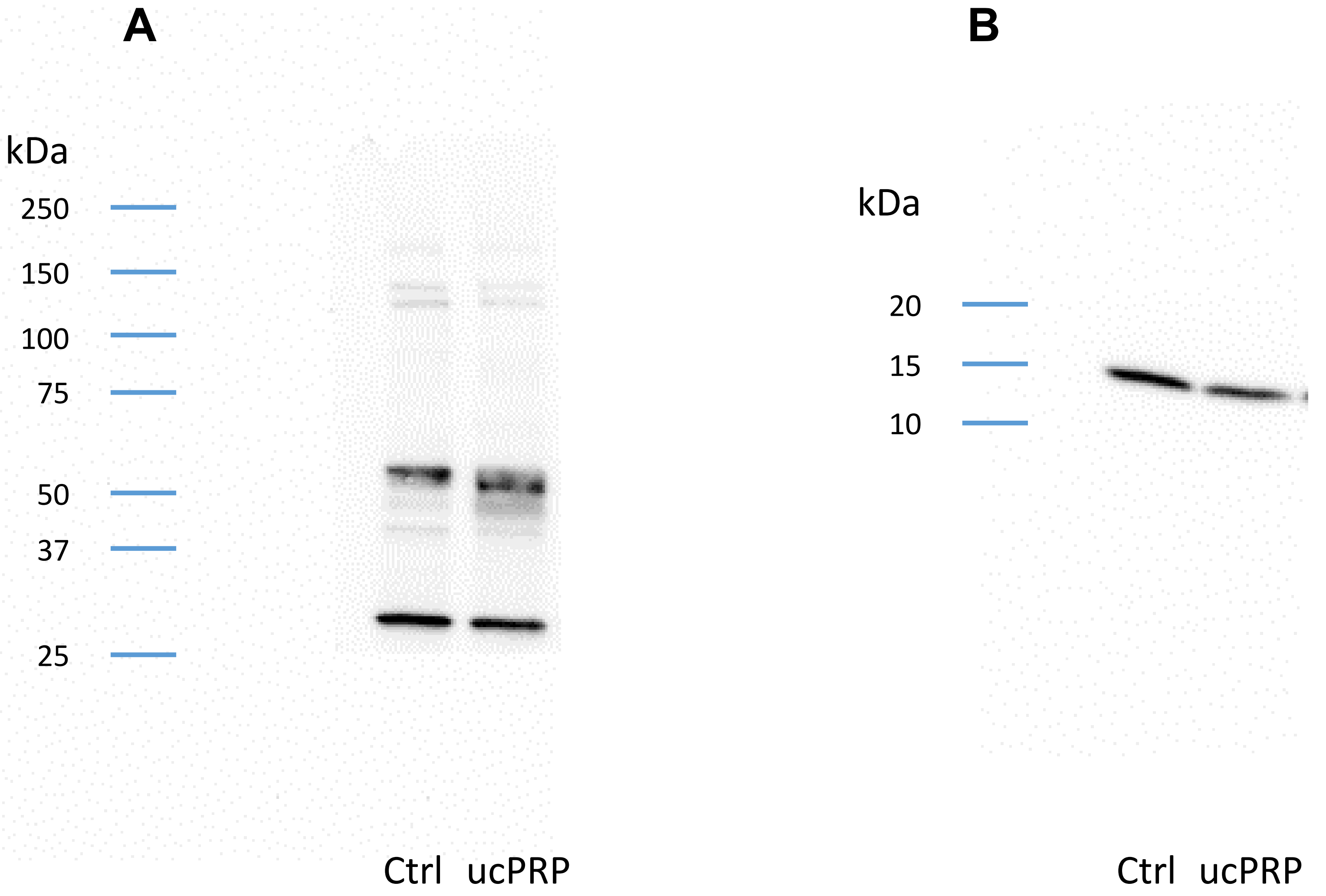

Figure 5 ORIGINAL IMAGES. ucPRP treatment increased the expression of Aquaporin-1 protein. (A) Representative western blots for aquaporin-1 (AQP1) and (B) β-2-microglobulin (B2M) in hCEC cells untreated (Ctrl) and treated with umbilical cord-derived PRP (ucPRP). AQP1 protein levels were assessed, revealing two major bands at approximately 28 kDa (monomer) and 56 kDa (dimer). Molecular weights markers are indicated on the left.
