# Supplementary material for: Umbilical Cord PRP Accelerates Corneal Wound Healing via AQP1 Upregulation and Calcium Signaling
Source: Biology (Basel). 2026 Apr 17;15(8):637. doi: 10.3390/biology15080637 (PMC13113299; doi:10.3390/biology15080637)
Supplement: Supplementary file 1 [file biology-15-00637-s001.zip › File S1. Orignal WB images/Figures S1 S2 original.pdf]

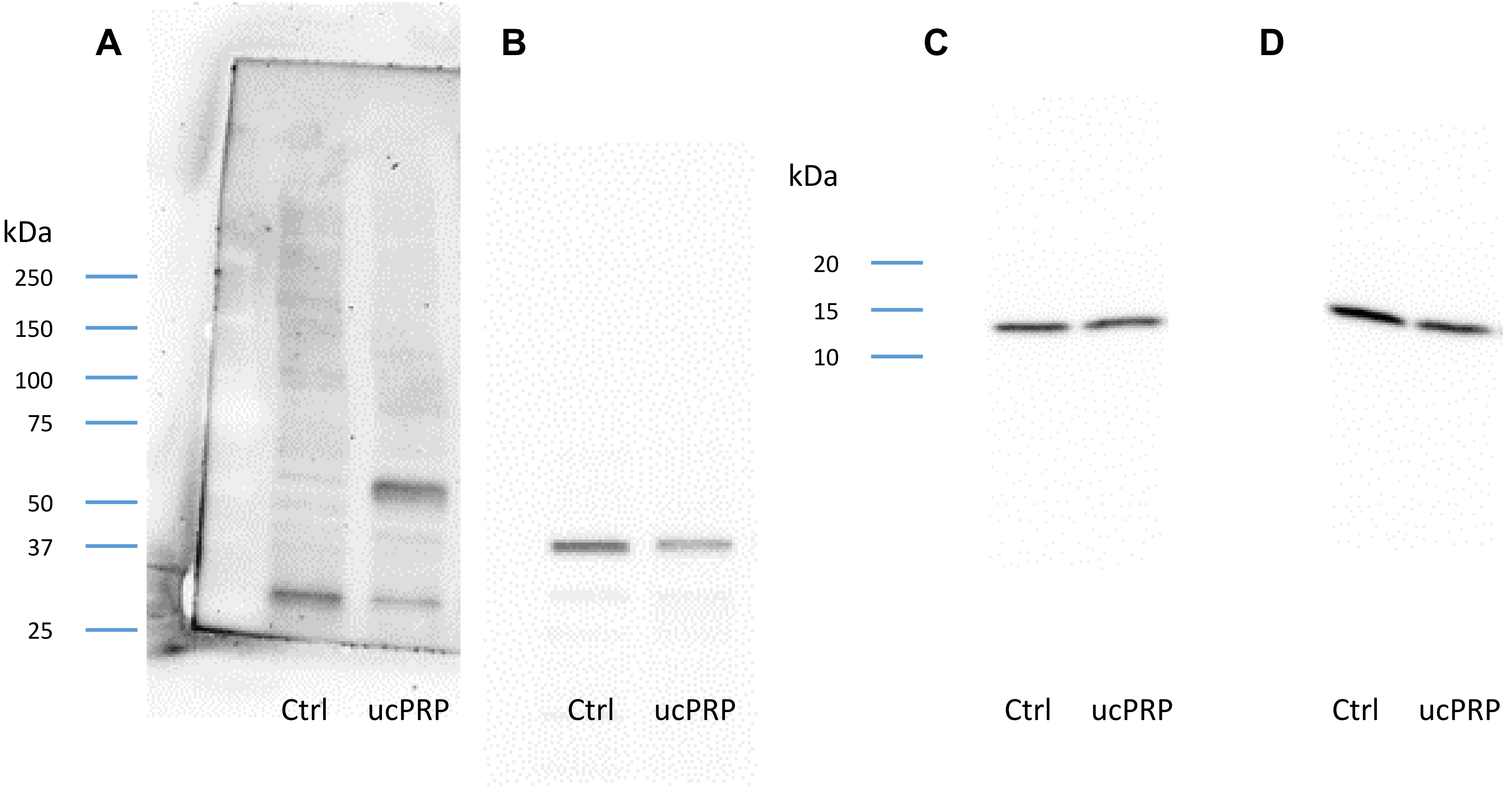


**Figure S1 ORIGINAL IMAGES.** Effect of ucPRP treatment on AQP3 and AQP4 protein expression. (A, B) Representative Western blot analyses of AQP3 (A), AQP4 (B), and (C,D) β2-microglobulin (B2M) in human corneal epithelial cells (hCECs) untreated (Ctrl) or treated with umbilical cord–derived platelet-rich plasma (ucPRP). (C) is the B2M of AQP3 and (D) the B2M of AQP4. Molecular weights markers are indicated on the left. For AQP3, bands correspond to the monomer (~29 kDa) and dimer (~58 kDa), while AQP4 is detected as a monomer at ~34 kDa.

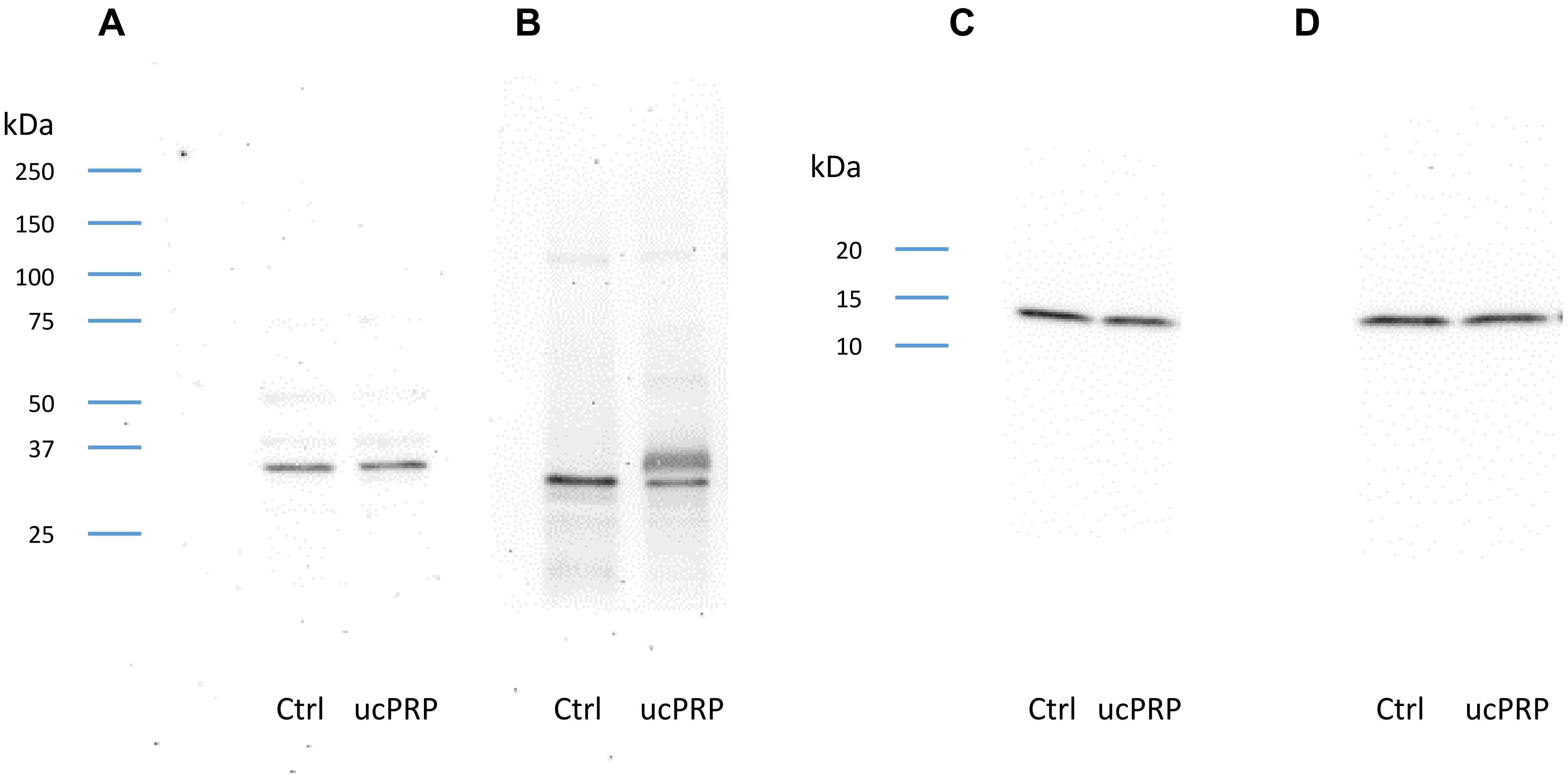


**Figure S2 ORIGINAL IMAGES.** Effect of ucPRP treatment on AQP5 and AQP9 protein expression. (A, B) Representative Western blot analyses of AQP5 (A), AQP9 (B), and (C,D) β2-microglobulin (B2M) in human corneal epithelial cells (hCECs) untreated (Ctrl) or treated with umbilical cord–derived platelet-rich plasma (ucPRP). (C) is the B2M of AQP5 and (D) the B2M of AQP9. Molecular weights markers are indicated on the left. For AQP5 and AQP9, bands are detected as a monomer at ~35 kDa and at ~33 kDa, respectively.
