# Supplementary material for: Umbilical Cord PRP Accelerates Corneal Wound Healing via AQP1 Upregulation and Calcium Signaling
Source: Biology (Basel). 2026 Apr 17;15(8):637. doi: 10.3390/biology15080637 (PMC13113299; doi:10.3390/biology15080637)
Supplement: Supplementary file 1 [file biology-15-00637-s001.zip › biology-4201284-supplementary-Figure S1_S2 Table S1.pdf]

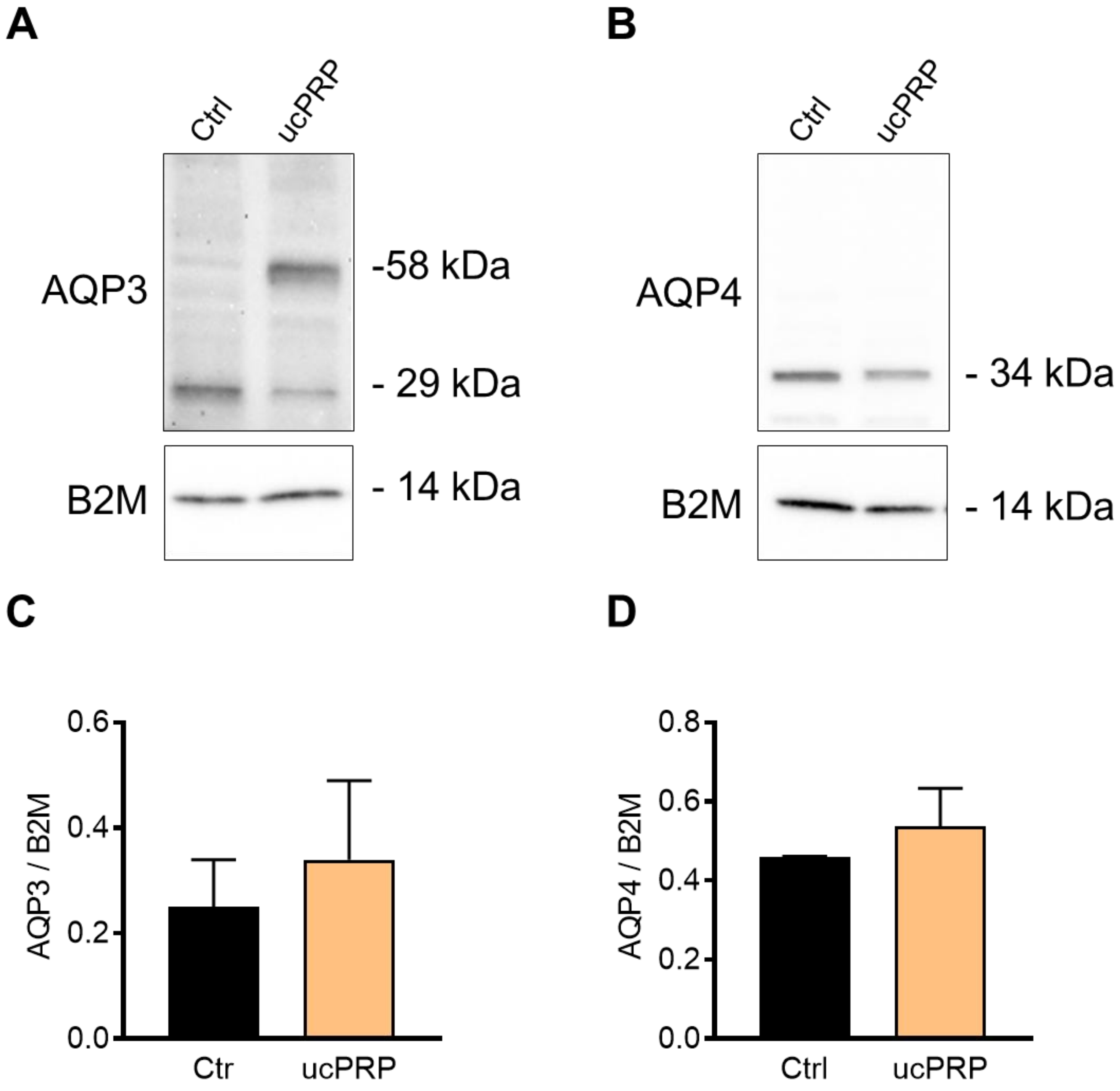

Figure S1. Effect of ucPRP treatment on AQP3 and AQP4 protein expression. (A, B) Representative Western blot analyses of AQP3 (A), AQP4 (B), and β2-microglobulin (B2M) in human corneal epithelial cells (hCECs) untreated (Ctrl) or treated with umbilical cord–derived platelet-rich plasma (ucPRP). Molecular weights are indicated on the right. For AQP3, bands correspond to the monomer (~29 kDa) and dimer (~58 kDa), while AQP4 is detected as a monomer at ~34 kDa. (C, D) Densitometric analysis of AQP3 (C) and AQP4 (D) Western blot bands was performed using iBA software (Thermo Fisher Scientific, Milan, Italy) and normalized to B2M expression. No statistically significant differences were observed between Ctrl and ucPRP-treated cells.

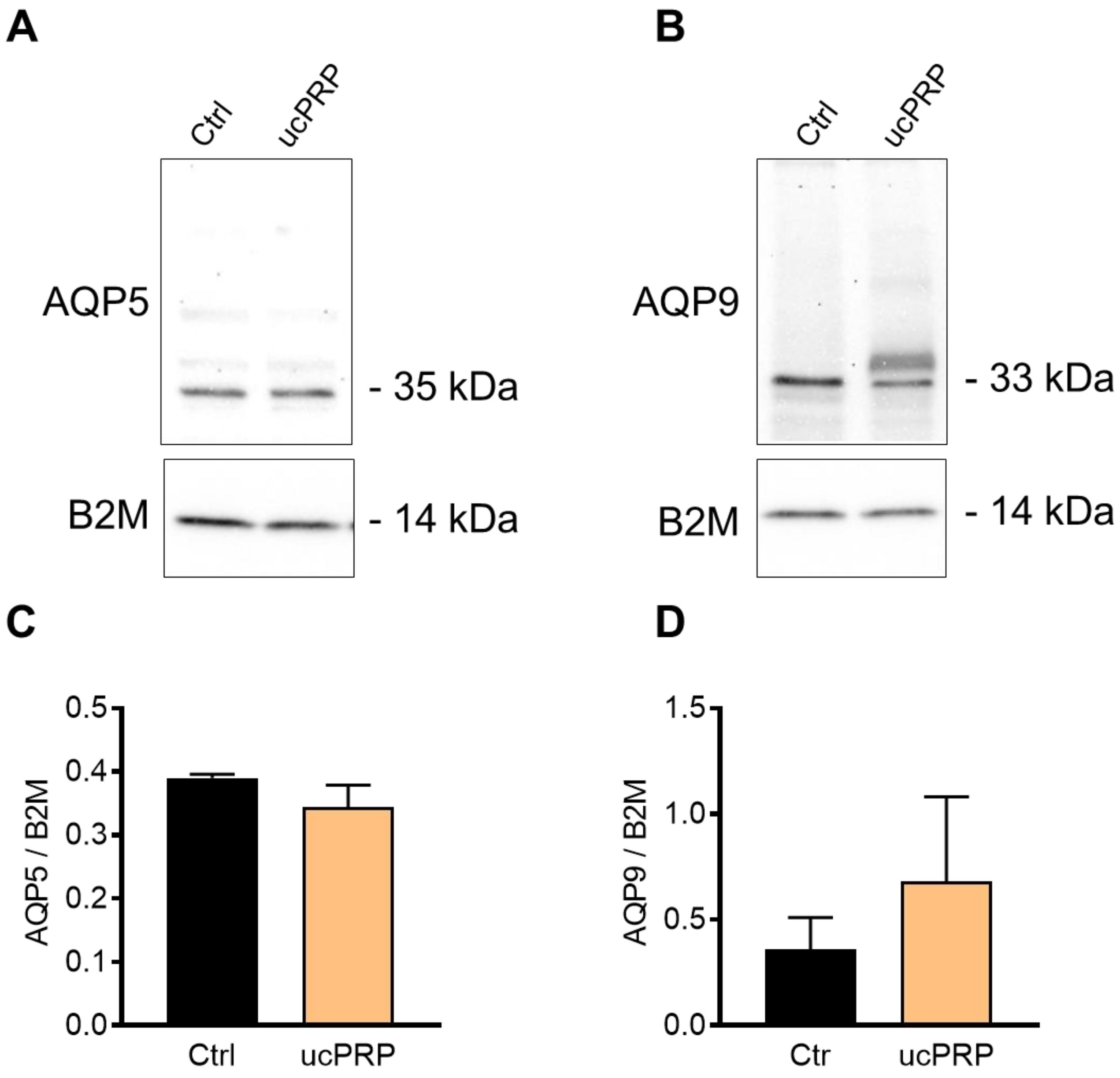

Figure S2. Effect of ucPRP treatment on AQP5 and AQP9 protein expression. (A, B) Representative Western blot analyses of AQP5 (A), AQP9 (B), and β2-microglobulin (B2M) in human corneal epithelial cells (hCECs) untreated (Ctrl) or treated with umbilical cord–derived platelet-rich plasma (ucPRP). Molecular weights are indicated on the right. For AQP5 and AQP9, bands are detected as a monomer at ~35 kDa and at ~33 kDa, respectively. (C, D) Densitometric analysis of AQP5 (C) and AQP9 (D) Western blot bands was performed using iBA software (Thermo Fisher Scientific, Milan, Italy) and normalized to B2M expression. No statistically significant differences were observed between Ctrl and ucPRP-treated cells.

Table S1. List of antibodies used in the study			
Antibody	Host	Source (catalog)	dilution
Anti-AQP1	Rabbit	St John's Laboratory Ltd, London, U.K. (STJ117224)	1:500
Anti-AQP3	Rabbit	BOSTER biological technology, California, U.S.A. (PA1488)	1:1000
Anti-AQP4	Rabbit	St John's Laboratory Ltd, London, U.K. (STJ22661)	1:500
Anti-AQP5	Rabbit	Merck, Milan, Italy (A4985)	1:500
Anti-AQP6	Rabbit	Alpha Diagnostic International, San Antonio, U.S.A. (AQP61-A)	1:1000
Anti-AQP7	Rabbit	Abcam, Cambridge, U.K. (ab32826)	1:500
Anti-AQP8	Rabbit	Merck, Milan, Italy (HPA046259)	1:1000
Anti-AQP9	Rabbit	Life Technologies Italia, Monza, Italy (PA5-97110)	1:500
Anti-AQP10	Rabbit	Abcam, Cambridge, U.K. (ab182794)	1:500
Anti-AQP11	Rabbit	BOSTER biological technology, California, U.S.A. (PB10044)	1:1000
Anti-B2M	Rabbit	Abcam, Cambridge, U.K. (ab75853)	1:10000
β-2-microglobulin, B2M.			
